# Host HLA B*Allele-Associated Multi-Clade Gag T-Cell Recognition Correlates with Slow HIV-1 Disease Progression in Antiretroviral Therapy-Naïve Ugandans

**DOI:** 10.1371/journal.pone.0004188

**Published:** 2009-01-14

**Authors:** Jennifer Serwanga, Leigh Anne Shafer, Edward Pimego, Betty Auma, Christine Watera, Samantha Rowland, David Yirrell, Pietro Pala, Heiner Grosskurth, Jimmy Whitworth, Frances Gotch, Pontiano Kaleebu

**Affiliations:** 1 MRC/UVRI Uganda Research Unit on AIDS, c/o Uganda Virus Research Institute, Entebbe, Uganda; 2 Department of Immunology, Imperial College, Chelsea & Westminster Hospital, London, United Kingdom; 3 Department of Medical Microbiology, Ninewells Hospital, Dundee, United Kingdom; University of California Los Angeles Medical Center, United States of America

## Abstract

**Background:**

Some HIV infected individuals remain asymptomatic for protracted periods of time in the absence of antiretroviral therapy (ART). Virological control, CD4 T cell loss and HIV-specific responses are some of the key interrelated determinants of HIV-1 disease progression. In this study, possible interactions between viral load, CD4 T cell slopes, host genetics and HIV-specific IFN-γ responses were evaluated in chronically HIV-1-infected adults.

**Methodolology/Principal Findings:**

Multilevel regression modeling was used to stratify clade A or D HIV-infected individuals into disease progression groups based on CD4 T cell slopes. ELISpot assays were used to quantify the frequency and magnitude of HIV-induced IFN-γ responses in 7 defined rapid progressors (RPs) and 14 defined slow progressors (SPs) at a single time point. HLA typing was performed using reference strand conformational analysis (RSCA). Although neither the breadth nor the magnitude of the proteome-wide HIV-specific IFN-γ response correlated with viral load, slow disease progression was associated with over-representation of host immunogenetic protective HLA B* alleles (10 of 14 SPs compared to 0 of 7; p = 0.004, Fisher's Exact) especially B*57 and B*5801, multiclade Gag T-cell targeting (71%, 10 of 14 SPs compared to 14%, 1 of 7 RPs); p = 0.029, Fisher's Exact test and evident virological control (3.65 compared to 5.46 log10 copies/mL in SPs and RPs respectively); p<0.001, unpaired student's t-test

**Conclusions:**

These data are consistent with others that associated protection from HIV disease with inherent host HLA B allele-mediated ability to induce broader Gag T-cell targeting coupled with apparent virological control. These immunogenetic features of Gag-specific immune response which could influence disease progression may provide useful insight in future HIV vaccine design.

## Introduction

Although there is urgent need for a protective human immunodeficiency virus (HIV) vaccine, the correlates of effective immune protection from HIV-1 infection remain unclear. RNA viral load and CD4 T-cell counts are the key markers of HIV-1 disease progression. The relationship between HIV-induced immune responses and virological control remains contentious. Inverse correlations between HIV-specific T cell responses and concurrent plasma viral load have been demonstrated by some investigators [1], [2], [3], [4] but could not be confirmed by others [5], [6], [7], [8], [9]. Furthermore, some studies reported discordant correlations between T-cell responses and viral load and demonstrated these relationships to be determined by the infecting clade, targeting of sub-dominant epitopes [10], region of HIV targeted [4], [11], [12], and disease status [13].

Some antiretroviral drug naïve HIV infected individuals remain asymptomatic for protracted periods showing relatively lower levels of plasma viral RNA and stable CD4 counts, and this beneficial state has been attributed to complex features associated with viral, host genetic and environmental factors. These features include slow or arrested viral evolution [14], [15], [16]; HIV subtype variation [17], [18]; a broadly directed T-cell response mostly targeting Gag [2], [9], [11], [19]; heterozygosity for the CCR5 Δ32 HIV receptor; enrichment of certain HLA haplotypes and HIV polymorphisms [20], [21], [22] and lack of immune activation [23].

True immune correlates of controlled HIV infection remain obscure. The cellular arm of the host immune system has been associated with partial virological control, remarkably demonstrated in studies of CD8+ T-cell depletion; CD8 T-cell immune escape and by the association between specific HLA class I alleles and favourable HIV disease outcome [21], [24]. Nevertheless these correlates are not absolute and, for example, a vaccinee who exhibited HIV-specific T- cell polyfunctionality with the appropriate memory phenotype, and targeting epitopes associated with long-term non-progression, became HIV infected [25]. Furthermore, an HIV vaccine based on the recombinant Adenovirus 5 (rAd5)-vector, showed good HIV-specific immunogenicity in Phase I studies using IFN-γ ELISpot assay, and exhibited long-lasting, multifunctional responses as monitored by polychromatic flow cytometry, but failed to protect Ad5-seronegative HIV acquisition in vaccinees with prior immunity to adenoviruses, reviewed in [26].

We used CD4 T-cell slopes to define HIV disease progression in a population of HIV infected, ART-naïve study participants in order to evaluate possible correlates of immune protection in HIV disease progression. We performed high resolution HLA typing, viral load estimation, CD4 T-cell quantitation and evaluation of HIV-induced IFN-γ responses to consensus HIV Gag, Nef, Rev, Vif, Tat, Pol, Vpr and Vpu peptides in order to investigate potential protective correlations at a single time point.

## Results

### Cohort stratification

Retrospective six-monthly CD4 T-cell counts were utilised in a multilevel regression model to stratify the cohort into HIV disease progression groups based on individual participant CD4 slopes. The median participant observation time from first CD4 count to recruitment into this study was 61 months (range 18–97 months). Using the model-derived CD4 slopes, 110 participants, 16 (15%) males and 94 (85%) females, were classified as rapid progressors (RP, n = 7, stratification 1), normal progressors (NP, n = 89, stratifications 2, 3 and 4) or slow progressors (SP, n = 14, stratification 5), illustrated in table 1. RPs were individuals with CD4 slopes steeper than −101 CD4 cells per µl/year; SPs had CD4 slopes which were rising (>16 CD4 cells per µl/year), while individuals with CD4 slopes between −91 CD4 cells per µl/year to +10 CD4 cells per µl/year were classified as normal progressors. These cut-offs were selected so as to be distinguishable; no participant had a CD4 decline between −101 and −91 CD4 cells per µl/year, nor between 10 and 16 CD4 cells per µl/year. Rapid progressors had a median annual CD4 T cell decline of 113 cells per µl/year (interquartile range [IQR] 116 to102 cells per µl/year). NPs had median annual CD4 T cell decline of 27 (IQR 51 to13) while SPs had median annual CD4 T-cell count rises of 24 cells per µl/year (IQR 20 to 46 cells per µl/year), table 1.

**Table 1:** Stratification of cohort into HIV-1 disease progression groupings.

No of Individuals	Range of CD4 counts utilised per person	Median annual CD4 count change (Interquartile range)		Cut-off CD4 T-cells per µl/year	Stratification group
		Undjusted^1^	Adjusted^2^		
7	8–18	−113 (−116 to −102)	−118 (−119 to −109)	<−**101**	**1**
8	10–17	−81 (−89 to −75)	−88 (−95 to −86)	−91 to +10	2
78	10–27	−24 (−47 to −13)	−38 (−58 to −29)		3
3	20–21	+10 (10 to 10)	−0 (−10 to −0)		4
14	14–24	+24 (20 to 46)	+9 (5 to 19)	>**16**	**5**

1 CD4+ T cell decline without adjusting for first CD4 count and age of the participant.

2 CD4+ T cell decline after adjusting for first CD4 count and age of the participant.

Table 1 illustrates the stratification of 110 chronically HIV-1 adults into distinct progression groups. Six-monthly retrospective CD4 counts were used in a multilevel regression model to derive individual participant CD4 slopes. The slopes were calculated over a median observation time of 610 months (minimum-maximum 18–97 months). Annual CD4+ T-cell changes are expressed as medians with interquartile ranges. Positive (+) symbols indicate increasing CD4+ counts while (−) indicates decreasing CD4+ counts over time. Individuals in the extreme stratification group 1 were selected as rapid progressors (RP, n = 7) while those in group 5 were selected as slow progressors (SP, n = 14). Groups 2, 4and 4 were categorised as normal progressors (NP, n = 89). Normal progressors were those with CD4 slopes between −91/year to +10/year; RPs had CD4 slopes <−91/year while SPs had CD4 slopes >+10/year.

### Study population demographics

The RPs and SPs evaluated in this study comprised of 19 females and 2 males with no significant difference in median age at recruitment between the two groups ([SP 37 years; interquartile range 30–43 years] and [RP 31 years; interquartile range 28–44 years]), table 2. All participants included in the data analysis were antiretroviral therapy naïve. Viral load data used in all analyses were computed as the mean of up to 3 six-monthly viral load measurements evaluated after recruitment into this study, table 1. The study participants were predominantly infected with HIV subtypes A and D, table 2.

**Table 2:** Study population demographics, host genetics and multi-clade gag recognition.

PTID	Annual CD4 slope	Gag IFN-γ				Infecting clade Determined from Gag	Age	Gender	Mean Log10vL	HLA-A	HLA-B	HLA-C
		A	B	C	D							
RP1	−114.1					D	30.5	F	5.77	A*36, A*74	B*45, B*72, Bw*06	Cw*2, Cw*6
RP2	−102.3					nd	41.5	F	5.90	A*02, A*68	B*07, B*45, Bw*06	Cw*7, Cw*16
RP3	−116.0					nd	26.5	F	6.34	A*02, A*74	B*08, B*45, Bw*06	Cw*7, Cw*16
RP4	−101.5					D	25.5	F	5.18	A*2301, A*6601	B*4501, B*5802, Bw*04, Bw*06	Cw*0602
RP5	−113.3				**+**	D	41.5	F	5.58	A*33, A68	B*42, B*49,	Cw*07, Cw*17
RP6	−116.4	**+**				A	43.5	F	4.84	A*3002, A*33	B*1510, B*5101/05	Cw*03, Cw*04
RP7	−106.1	**+**			**+**	nd	28.5	F	4.65	A*3004/06, A*8001	B*1401, B*1801/03/05/08,	Cw*0202, Cw*0302/06
SP1	+43.7					nd	28.5	F	3.96	A*3401, A*6601	B*4403, B*8101, Bw*4/*6, Bw*6	Cw*0401, Cw*0701
SP2	+16.3	**+**	**+**	**+**	**+**	D	39.5	F	4.97	A*0201/*0209, A*2301	B*0801, B*4501 Bw*06	Cw*10, Cw*16
SP3	+22.0	**+**		**+**	**+**	D	48.5	F	3.79	A*2902	B*57, B*5801	Cw*04, Cw*07
SP4	+37.7					A	33.5	M	2.69	A*02, A*30	B*57, B*58	Cw*04, Cw*06
SP5	+26.1	**+**			**+**	A	35.5	F	4.18	A*6601, A*7401/*7402	B*5301, B*5802, Bw*4, Bw*6	Cw*0401, Cw*0602
SP6	+20.9		**+**	**+**		D	29.5	F	4.52	A*2301, A*3601	B*5301, B*3910, Bw*4, Bw*6	Cw*04, Cw*12
SP7	+77.2	**+**		**+**		A	41.5	F	1.65	A*0202, A*3003/*3003	B*4501, B*5801, Bw*4, Bw*6	Cw*0701, Cw*1601
SP8	+16.4	**+**			**+**	D	55.5	F	3.19	A*0201, A*6802	B*63, B*72	Cw*02, Cw*14
SP9	+22.7	**+**	**+**	**+**		D	36.5	F	3.64	A*0101, A*0301	B*4415, B*5701, Bw*4	Cw*0701, Cw*1801
SP10	+22.6	**+**	**+**	**+**	**+**	nd	37.5	F	1.65	A*3002, A*7401/02/03	B*49, B*5703	Cw*0701/05/06
SP11	+49.1					nd	25.9	F	3.97	A*0101, A*3001	B*4201, B*5801, Bw*4/*6, Bw*6	Cw*10, Cw*17
SP12	+44.8		**+**	**+**		D/B	31.1	F	3.78	A*3001, A*6602	B*1801, B*67, Bw*1	Cw*0704, Cw*1203
SP13	+19.0	**+**	**+**	**+**		A	34.5	M	4.20	A*0201/*0209, A*3002/*3003 A*30	B*5701, B*72, Bw*4, Bw*6	Cw*0202, Cw*1801
SP14	+54.5					nd	42.2	F	4.95	A*0201/*0209	B*4201, B*4501, Bw*6	Cw*1601, Cw*1701

Table 2 illustrates study participant demographics and IFNγ response to clades A, B, C and D gag peptides. CD4+ T –cell slopes were derived from multilevel regression analysis of retrospective 6-monthly CD4+ T-cell counts. Under annual CD4 slope, symbol (−) indicates model-derived decreasing CD4 slopes while (+).indicates decreasing CD4 slopes over time. Under Gag-induced IFN-γ, areas marked + indicate induction of Gag-induced IFN-γ responses to the respective clade of Gag peptides, blank areas indicate lack of IFN-γ response. Clade of the infecting HIV virus was determined from partial sequences of the Gag region, “nd” indicates not done. Bold highlights indicate HLA alleles that have been reported to confer protection from HIV disease.

### Comparison of plasma viral loads

**Figure 1:**
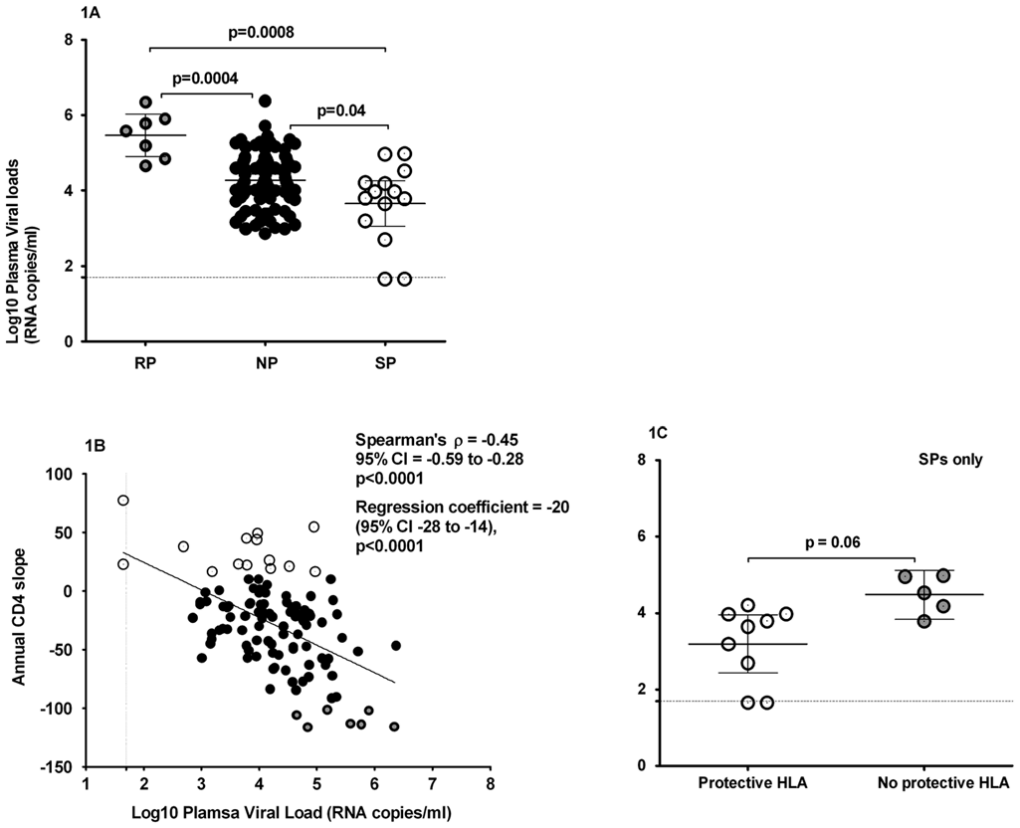
Evaluation of relationship between HIV disease progression and viral loads. CD4 slopes were computed by multilevel regression modelling of six-monthly retrospective participant CD4 counts. The median observation time over which CD4 slopes were calculated was 61 months (minimum 18 and maximum 97) months. Plasma viral load was quantified using Bayer™ bDNA assay according to the manufacturer's protocol; the plasma viral load minimum detection threshold was 50 RNA copies/ml. Figure 1A illustrates the relationship between CD4 slopes and viral loads, ○denotes RPs, • are NPs; while ⊙are SPs; 1B compares mean viral loads between groups while figure 1C compares plasma viral loads in SPs who had HLA alleles known to be protective as previously described and to those who did not. The lines running parallel to the Y and X axis (figures 1A and 1B respectively) indicate the minimum detection limit for evaluation of plasma viral loads. For purposes of statistical analyses and graphical representation of the data, undetectable plasma viral loads were assumed to equate to 45 RNA copies per ml.

We evaluated the relationships between HIV-1 disease progression and plasma HIV RNA viral loads as these are key factors known to be associated with HIV-1 disease progression. The mean log10 plasma viral loads over the entire observation period were significantly higher in RPs (5.46 log10 copies/mL) compared to SPs (3.65 log10 copies/mL); p<0.001, Students t-test, figures 1a. These data support virological control as one of the correlates of protection from HIV disease progression.

### Relationship between CD4 slopes and plasma viral loads

We evaluated the data for the relationship between CD4^+^ T-cell slopes and plasma viral loads, figure 1b. In the whole cohort, there was an inverse correlation between CD4^+^ T-cell slopes and plasma viral loads (Spearman's ρ = −0.45; p<0.0001, Spearman rank correlation test). An overall cohort regression coefficient of −20 (95% CI −28 to −14); p<0.0001implied that each log10 increment in plasma RNA load accounted for an annual loss of 20 CD4^+^ T-cells/µl in this cohort. When RP and SP were evaluated together excluding the NPs, each log 10 increment in plasma viral load accounted for an annual loss of 37 CD4^+^ T-cells/µl (95% CI −54 to −20 ); p<0.001. We were unable to evaluate RP or SP independently due to sample size limitations. These data implied that trends in CD4^+^ T-cell slopes were partially influenced by the levels of circulating virus.

### Participant host genetics

We evaluated the relationship between the host HLA alleles and HIV disease progression in this cohort. Slow progressors possessed significantly more of any of the following previously reported protective HLA B alleles B*27, B*57, B*5801, B*63, B*13, B*44, B*39 or B*81 (10 of 14) compared to RPs (0 of 7); p = 0.004, Fisher's Exact test, table 2. Most of these protective HLA were attributed to enrichment with HLA B*5801 and B*5701/*5703 alleles (including B*63 which is known to present similar epitopes as those presented by B*57). These alleles occurred at a frequency of 8 of 14 SPs compared to 0 of 7 RPs; p = 0.018, Fisher's Exact test, table 2. These data imply an association between slow HIV-1 disease progression and host immunogenetic determinants characterised by overrepresentation of protective HLA B alleles.

### Virological factors

The infecting clade of HIV-1 was determined from partial sequences of the Gag p17 and p24 Gag region and this cohort was found to be mainly infected with HIV-1 clades A or D virus, table 2. We investigated the degree of diversity or signature sequences within RPs but absent in SPs. This evaluation was limited by the small section of Gag sequence and the small number of RPs sequences which were mainly subtype D. Where we could make comparisons using the incomplete Gag sequence data, there was no apparent correlation between viral sequence diversity and HIV disease progression (data not shown). We could not ascertain if there were other Gag regions with protective or unfavorable signature sequences.

Because SPs were enriched with protective B*57 and B*5801 alleles, we used the available sequence data to evaluate the presence of Gag mutations known to impair virus replication and fitness, table 3. Both T242N and A163G substitutions in the TSTLQEQIAW (TW10; gag 240–249) and KAFSPEVIPMF (KF11; Gag 162–172) Gag epitopes respectively, and known to be associated with impaired virus replication and fitness were observed in three of the four B*57/*5801 SP participants, table 3. These data indicate that slow disease progression was associated with a host genetic mechanism characterised by HLA-mediated immune pressure coupled with apparent impairment of viral replication.

**Table 3:** Evaluation of sequence data for Gag substitutions known to impair viral fitness.

Gag 162–172 A163G substitution		Gag 240–249 T242N substitution		Protective HLA
KAFSPEVIPMF		TSTLQEQIAW		
RP1	KAFSPEVIPMF	RP1	TSTLQEQX	
RP2	KAFSPEVIPMF			
		RP4	TSTLQEQI	
		SP2	TSTLQEQI	
SP3	KAFSPEVIPMF	**SP3**	TS**N**LQEQI	B*57, B*5801
SP5	KAFSPEVIPMF	SP5	TSTLQEQV	
SP6	KAFSPEVIPMF	SP6	TSTLQEQV	B*3910
**SP7**	K**G**FSPEVIPMF			B*5801
SP8	KAFSPEVIPMF	SP8	TSTP----	B*63
SP9	RAFSPEVIPMF			B*5701
**SP13**	R**G**FSPEVIPMF	SP13	TSTPQEQM	B*5701

Table 3 illustrates the sequence variation within the HLA B5701 and B^*^580-restricted p17Gag 162–172 KAFSPEVIPMF (KF11) and p24Gag 240–249 TSTLQEQIAW (TW10) epitopes. Bold and underlined letters indicate substitutions in these Gag epitopes known to incur significant reduction in viral replicative capacity following immune pressures. Only known protective HLA alleles are indicated. Only available sequence data is presented.

### Characterisation of HIV-induced IFN-γ responses

**Figure 2:**
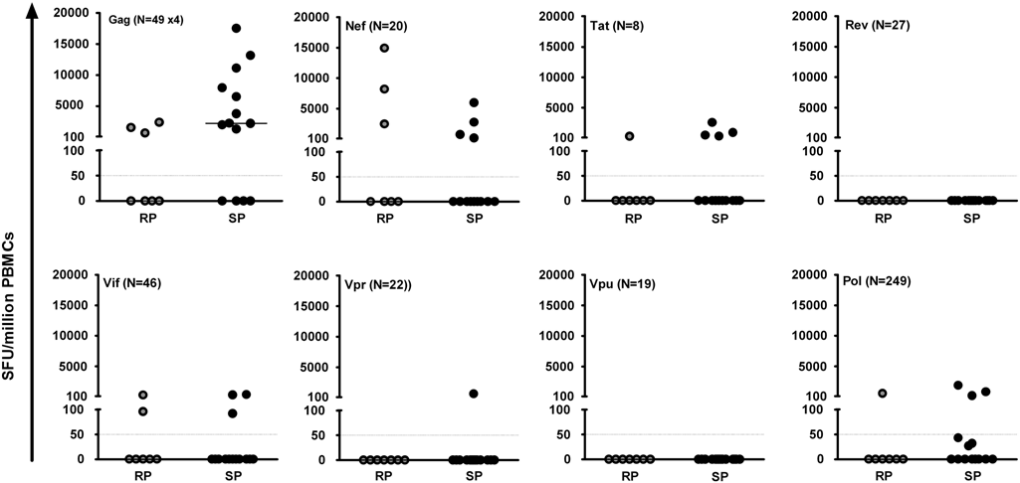
Relationship between HIV-induced IFN-γ magnitudes and disease progression. Figure 2 illustrates the total magnitudes of HIV-induced IFNγ responses against clades A, B, C and D Gag; and clade B Nef, Tat, Rev, Vif, Vpr, Vpu and Pol proteins. Choice of clades of peptide evaluated was dictated by availability from the NIH reagent programme. It is likely that the total IFN-γ response to Nef, Tat, Rev, Vif, Vpr, Vpu and Pol would increase if peptide sets from all four clades had been used. The primary purpose of these graphs is to compare responses induced between SP and RP. This goal is achieved even though Gag will evidently have a higher response rate as all four clades are used, as opposed to only clade B for other regions. The cut off for a positive response was at least 50 SFU/million PBMCs after subtracting off twice the mean background. Data is presented as net response after subtracting off all backgrounds. All net responses below the cut-off have been equated to zero response. Horizontal lines inside the graphs indicate cut-off points for a positive response. Horizontal bars around the data points indicate medians, Note that when more than 50% of the data is zero, the median is zero therefore horizontal bars are missing where medians were zero. N indicates the total number of peptides analysed for that particular HIV protein, note that different numbers of peptides per protein contribute these observed magnitudes.

**Figure 3:**
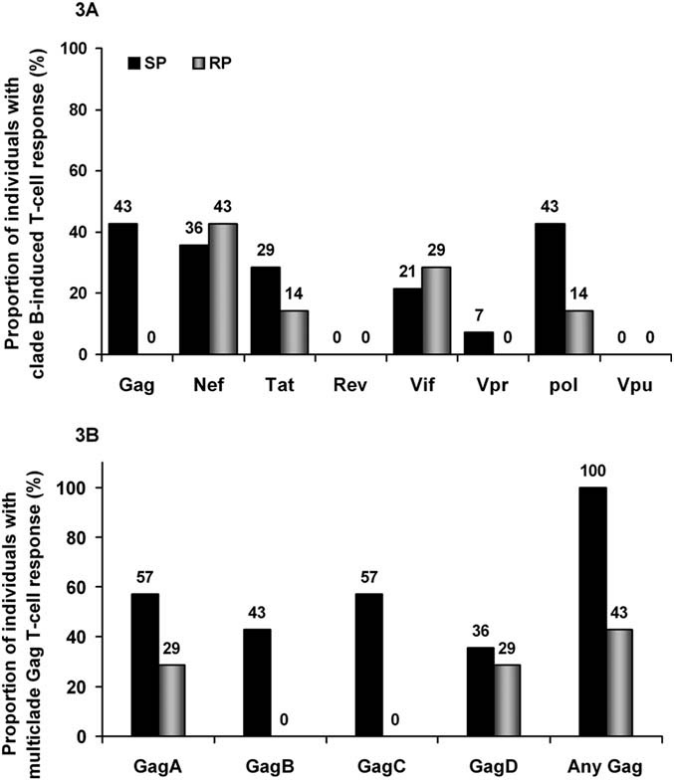
Breadth of HIV-induced IFN-γ T-cell recognition. The peptides sets evaluated were dictated by availability from the NIH AIDS reagent programme; clades A, B, C and D Gag peptides were obtainable while only clade B was available for Nef, Tat, Rev, Vif, Vpr, Pol and Vpu proteins. Figure 3 compares the proportion of participants (%) inducing HIV-specific IFN-γ responses using clade B peptides only, figure 3A; as opposed to multiple clade Gag peptide sets, figure 3B.

We evaluated the relationship between the HIV-induced Gag, Nef, Tat, Vif, Rev, Vpr, Vpu and Pol T-cell recognition and HIV disease progression; figures 2 and 3a. Overall, HIV specific T-cell responses were detected against Gag, Nef, Tat, Vif and Pol proteins in a proportion of both RP and SP; Vpr responses were seen only in a single SP while there were no detectable responses to Rev or Vpu in any RP or SP participant. Overall, the median magnitudes of response did not significantly differ between RPs and SPs, although total Gag-induced responses tended to be higher in SPs (median 2253 SFU/million PBMCs, IQR 0 to 8756) compared to RPs (median 70 SFU/million PBMCs, IQR 0 to 1552); p = 0.095, Kruskal Wallis test. These data suggest that slow HIV disease progression was associated with preferential Gag T-cell recognition in this cohort.

### Characterisation of Gag-induced T-cell recognition

**Figure 4:**
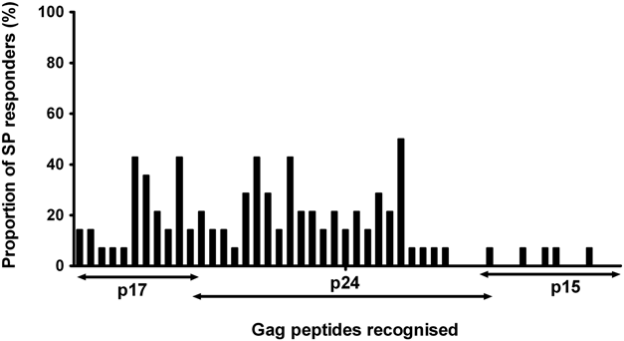
Distribution of Gag T-cell recognition among slow progressors. Figure 4 illustrates the frequency of Gag T-cell recognition across the p17, p24 and p15 Gag region among the slow progressors. The frequency is presented as the proportion of SP individuals with Gag T cell recognition. The horizontal axis represents the peptides recognised.

The consensus peptides evaluated in this study were determined by availability from the NIH reagent repositories. Only peptides representative of clade B consensus were available, except for the Gag region for which Clades A, C and D consensus peptides were additionally obtained. We evaluated how the repertoire of consensus Gag peptide sets used influenced quantification of HIV-1 specific immune responses. Overall, use of four peptide sets detected significantly broader responses; six more participants showed Gag T-cell recognition when stimulated with four sets of Gag peptides compared to when only clade B peptides were evaluated, (13 of 21 responders compared to 7 of 21 respectively), table 2 and figure 3b. These T-cell responses principally targeted p17 and p24 Gag regions, figure 4. Taking into account the sequence lengths, there was no significant difference between targeting of the Gag p17 and p24 regions.

**Figure 5:**
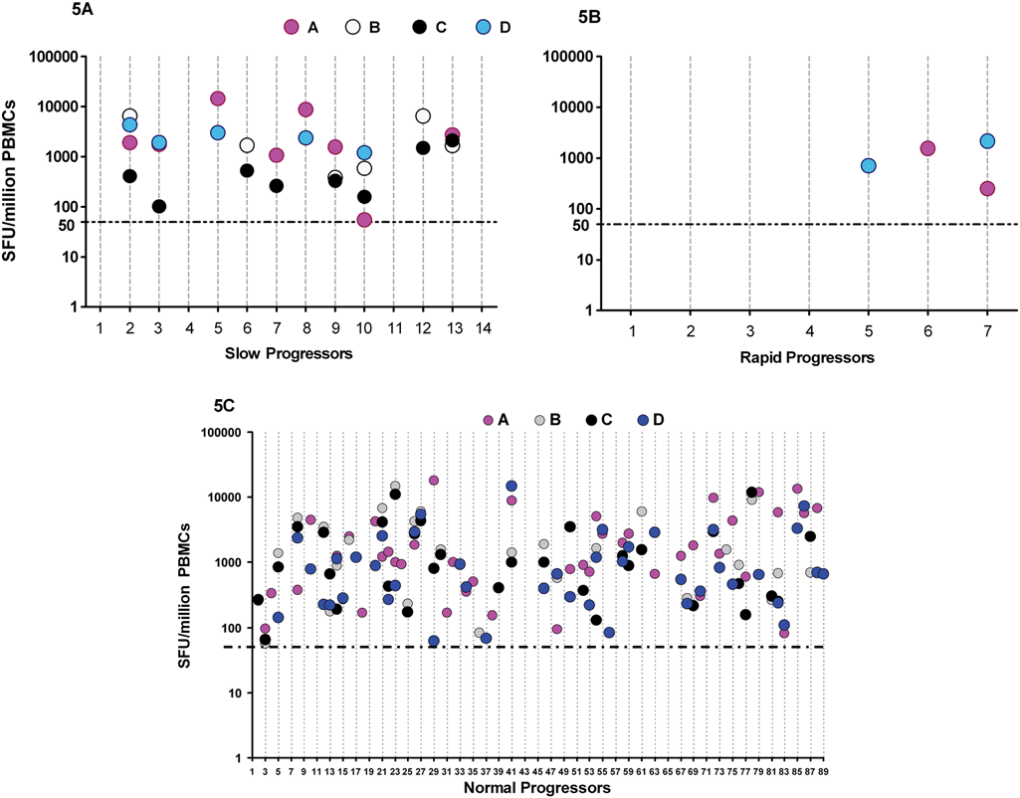
Relationship between multiclade Gag T-cell recognition and HIV disease progression. Figure 5 illustrates the total magnitudes of HIV Gag-induced IFNγ responses evaluated using ELISpot assay. A test was considered positive when response was ≥50 SFU/million PBMCs and at least twice the mean background response (6 wells of cells and media response only). The data is presented as net response; all background values have been subtracted. For purposes of statistical analysis and graphical representation, all negative responses (less than 50 net SFU/million PBMCs) were equated to zero SFU/ml PBMCs. Because the Y axis is presented in log, negative responses are not represented on these graphs. The X-axis represents individual participants. The horizontal dotted lines parallel to the X-axis represent the cutoff for a positive response. Slow progressors (SPs) are presented in figure 5A; rapid progressors (RPs) are in figure 5B while normal progressors are in figure 5C.

Overall, multi-clade Gag T-cell recognition was observed in 62% (61 of total 99) of the study participants (data not shown). Eleven participants were excluded from this analysis because they did not have data on all four Gag clades. These multi-clade responses were of higher magnitude and significantly more common among SPs (71%, 10 of 14) and NPs (64%, 58 of 78) compared to RPs (14%, 1 of 7), figures 5a, 5b and 5c; p = 0.029, Fisher's Exact). The frequency of multi-clade Gag T-cell recognition was not significantly different between SPs and NPs. While both SPs and RPs recognised Clades A and D Gag peptides, only SPs exhibited cross reactivity to the non-endemic clades B (6 of 14 ; p = 0.06, Fisher's Exact) and C (8 of 14; p = 0.018, Fisher's Exact), table 2. These findings suggest that slow disease progression was associated with broader multi-clade Gag T-cell recognition characterised by preferential targeting of the p17 and p24 regions. The data also indicate that quantitation of HIV-induced T-cell responses is influenced by the number of different consensus HIV peptide sets used.

### Relationship between IFN-γ response, host HLA alleles and plasma viral loads

We evaluated the relationships between the HIV-induced IFN-γ responses and plasma viral loads. Surprisingly, while SPs had significantly higher Gag-induced IFN-γ responses and significantly lower viral loads, we did not find a statistically significant relationship between IFN-γ responses and plasma viral load. However, the sample size evaluated in this cohort was small and this could have limited the reliably of the statistical evaluation.

Because SPs had significantly more representation any of the previously described protective HLA B alleles, we assessed SPs for the relationship between possession of these protective HLA B alleles (B*57, B*5801, B*63, B*13, B*44, B*3910, B*8101) and plasma viral loads. Slow progressors with known protective HLA B alleles had borderline significantly lower mean plasma viral loads (3.33, 95% CI 2.60 to 4.06) compared to SPs who lacked those B alleles (4.47, 95% CI 3.53 to 5.41); p = 0.060, Student's t-test, figure 1c. These data suggest that the apparent virological control observed in slow disease progression was partly mediated through inherent host immunogenetic mechanisms.

## Discussion

The main objective of this study was to characterise correlates associated with protection from HIV-1 disease progression as determined by CD4+ T cell slope. The CD4 slope which represents changes in numbers of CD4 T-cells over time strongly predicts HIV disease progression; an annual CD4 depletion of 10 additional cells/µl has been associated with a 2% increased likelihood of developing AIDS [27], [28]. We used CD4 T cell slopes to stratify cohort participants into distinct HIV disease progression groups in order to investigate possible relationships with protection. CD4 T-cell slopes in individual participants were estimated using a multilevel regression analysis model that predicted CD4 trends over time. Because the distinction between RP, NP, and SP was determined based on progressive CD4 changes, individual CD4 slopes were dispersed in a continuum distribution where the difference between adjacent groups would not be significant but there would be an expected trend that produces significant differences between the extreme groups (RPs and SPs). For example, variables in participants at the slow end of the NP would not be expected to be significantly different from those of the neighboring SPs. Consequently, the two extreme groups with CD4 slopes markedly different from each other were selected for more detailed evaluation of possible correlates of protection from HIV disease progression. Based on the multilevel model-derived stratifications, variables like plasma viral loads and protective HLA alleles were also demarcated with significant differences between the extreme groups. It is important to note that HLA typing in this study was performed after all other experiments and there was no pre-selection. These data indicate that in the absence of known dates of infection, CD4 slopes can be used to stringently delineate participants into distinct disease progression groups.

Evaluation of HIV-induced IFN-γ responses in this study was based on recognition of peptides derived from consensus HIV-1 sequences. The clades of peptides used were limited by availability through the NIH reagent repository; clade B peptides were used for all HIV regions except Gag for which Clades A, C and D were available. Where we could make comparisons on the limited sequence data (data not shown), there appeared no obvious consistent sequence variation between the RPs and SPs and the consensus, that is, within-group variation was not significantly different from between-group variation in similar HIV-1 clades implying that differences in disease progression were not attributable to viral diversity.

The use of consensus peptides to evaluate HIV-induced responses may have underrepresented the possible extent of the response repertoire. Furthermore, use of clade B peptides in a clade A or D infected population may have additionally underestimated the eventual breadth of responses. This was evidenced by the fact that six additional Gag responders were detected when multiple peptide sets were evaluated compared to when clade B peptides were analysed alone. Detection of more responses using four consensus peptide sets compared to one peptide set signifies how use of different peptide sets affects T-cell response quantification and highlights the importance of maximizing coverage of HIV-1 sequence diversity when evaluating CTL responses in HIV-1-infected individuals. These findings add to other studies that highlighted limitations arising from evaluation of HIV-1 induced immune responses using restricted repertoires of peptide sets [2], [29].

We found HIV-specific IFN-γ responses to Gag, Nef, Tat, Pol, Vif and Vpr in both RPs and SPs, but not to Rev and Vpu in this population. These responses did not significantly differ between RPs and SPs except for Gag-induced responses that tended to be higher in SPs compared to RPs suggesting a possible relationship between targeting of Gag and slow HIV-1 disease progression. These findings are consistent with previous findings in a clade C HIV-1 chronically infected cohort where only Gag responses but not Rev, Tat, Vif, Vpr, Vpu and Nef were associated with protection [11]. Furthermore, when RPs, NPs and SPs were compared, there was significantly more Gag T-cell recognition among SPs and NPs compared to RPs. Higher frequencies and magnitudes of Gag-induced responses in SPs compared to RPs suggests a beneficial relationship between targeting of Gag and slow HIV-1 disease progression, and add to several previous studies that have previously reported associations between HIV-1 Gag-specific T-cell targeting and protection [2], [3], [9], [11], [30], [31], [32].

Divergence of HIV-1 into multiple clades poses a worldwide challenge for HIV vaccine development. Demonstration of T-cell cross recognition of epitope sequences from different clades may offer hope for a global vaccine. Interpreted from IFN-γ ELISpot reactivity to the multiclade peptide sets, cross-clade immune recognition has been previously reported in several African populations [33], [34], [35], [36], [37], [38], but the relationship between these responses and HIV disease progression has not been previously described. Based on the previous assumptions that ELISpot reactivity to the multiclade Gag peptide sets predicts recognition of virally-infected cells, we found slow disease progression to be significantly more associated with multi-clade Gag T-cell recognition; SPs and NPs had significantly higher multiclade Gag T-cell recognition compared to RPs suggesting a beneficial relationship between Gag T-cell targeting and slow disease progression. We did not find significant difference in Gag multi-clade T-cell recognition between SPs and NPs, this is probably explained by the fact that the stratification into HIV disease progression groups was based on a continuum of CD4 slopes which was not expected to significantly demarcate between adjacent groups but would result in a considerable difference between the extreme RPs and SPs.

However, these multi-clade cross reactivity findings need to be interpreted with caution taking into consideration two recent studies that have questioned the validity of using single concentrations of peptides in ELISpot and intracellular cytokine staining assays for assessing cross-clade CTL activity by showing that cross clade reactivity determined by these assays does not always predict recognition of virally-infected cells [39], [40]. Evaluations of functional avidity of the epitope-specific T cells and/or actual ability to inhibit viral replication in vitro will be necessary to properly elucidate the fine specificities of these cross-reactive responses which was not established in this study due to limitations in cell numbers.

Inverse correlations between HIV-induced IFN-γ responses and plasma viral load have been reported by some investigators [3], [41], but could not be confirmed by others [5], [7]. Studies that demonstrated inverse correlations between IFN-γ responses and plasma viral loads related this to a broader HIV-1 epitope repertoire [9], [42] which in this study could have been limited by the use of peptides based on consensus sequences instead of autologous sequences. Others attributed virological control to the ability to induce HIV-specific polyfunctional responses but not to total HIV-specific CD8+ T-cell IFN-γ frequencies or magnitudes implying that rather than quantity or phenotype, the quality of the CD8(+) T-cell functional response correlated with protection from HIV disease progression [43]. In this study, we found no correlation between HIV-induced IFN-γ responses and plasma viral loads; however such statistical evaluation was limited by the small sample size of our cohort.

Despite the previously reported relationships between polyfunctional T-cell responses and protection from HIV-1, clarification of correlates of protection remains elusive. More recent studies have demonstrated that spontaneous control of viraemia may occur even in the absence of highly polyfunctional CD8+ T cell responses [44], while others found no major difference in T-cell polyfunctionality between rapid and slow progressors [45], [46], [47] implying that the correlates of protection from HIV disease are not readily discernible with the standard assays used to measure immune responses. In the current study, four slow progressors who lacked measurable Gag IFN-γ responses had marginal IFN-γ responses to Pol, Tat or Vpr (data not shown) further implying that the IFN-γ producing T cells may not be the only relevant subsets; factors other than IFN-γ may have contributed to the observed slow disease progression. Three of these four SPs possessed HLA B*57 or B*5801 or B*8101 that have been demonstrated to be associated with virological control implying possible involvement of a host HLA associated cellular immune mechanism other than IFN-γ investigated here.

Evidence for qualitative differences in protective HIV-induced T cell responses arise from immunogenetic studies that have revealed associations between expression of particular alleles such as HLA-B*57, B*5801, B*8101 and B*27 with successful virological control [16], [48], [49], [50], [51] and associations between expression of other alleles such as HLA B*5802, B*18 and HLA B*3502/03 with unsuccessful control of HIV [21], [52], [53]. The host's genetic background plays a vital role in the evolution and immune control of HIV-1 infection and expression of particular HLA B alleles has been reported to have the strongest influence on viral load, CD4 count and selection pressure on the virus [50]. Several reports have demonstrated over representation of HLA B*27, and B*57/*5801 alleles with slow HIV disease progression; HLA*B63 has been demonstrated to confer protection through presentation of epitopes similar to those presented by HLA-B*57, while others such as HLA-B*13, HLA B*44, HLA B*39 and HLA-B*81 alleles have also been associated with protection [2], [21], [22], [48], [49], [50], [51]. In contrast, HLA B*5802 and B*18 have been linked to high viraemia [52], [53]. Despite the limited sample size in this study, we observed an over representation of previously reported protective HLA B alleles especially B*57/B*58 within this cohort. Furthermore, slow progressors who had protective HLA B alleles had lower plasma viral loads than those who did not implying a host HLA driven beneficial relationship. This apparent differential distribution of protective HLA profiles according to level of viraemia suggests an important host genetic and/or immunologic mechanism to protection from HIV disease progression in this population.

HLA B57/B58-driven immune pressure on two Gag epitopes has been demonstrated to result in immune escape with consequential virus fitness cost. Several previous reports have demonstrated that selection pressure on Gag epitopes TSTLQEQIAW (TW10; gag 240–249) and KAFSPEVIPMF (KF11; Gag 162–172) induces immune escape through T242N and A163G substitutions respectively, resulting in impaired viral replication and reduction in virus fitness [21], [41], [54], [55], [56], [57]. Others have linked the beneficial clinical outcome of enrichment of HLA B57/5801 in long term non-progressors to impaired HIV-1 replication capacity rather than differences in CTL escape mutations or CTL activity against epitopes in Gag [16]. The mechanism for virological control was not fully established in this cohort. However, there was evidence of immune escape in the KF11 and TW10 Gag epitopes with substitutions known to be associated with impaired virus fitness in three of the four evaluated B*57 slow progressors partly explaining the apparent virological control observed in these individuals.

Taken together, these findings suggest that slow HIV-1 disease progression in this cohort was associated with a host immunogenetic mechanism that was partially mediated through preferential targeting of Gag and intrinsic immunogenetic HLA B-driven immune pressure in critical Gag regions as well as host genetic associated relative control of HIV replication.

These data are consistent with data from studies that have related HLA-class I alleles and targeting of multiple Gag epitopes with relative suppression of viraemia, and have implications for HIV-1 vaccine development.

## Materials and Methods

### Study population

110 study participants were recruited from a Medical Research Council (MRCUK)-funded, HIV-infected “prevalence” cohort attending The AIDS Support Organisation (TASO) HIV counseling and care services, in Entebbe, Uganda. This cohort is largely infected with HIV-1 clades A and D and is predominantly female. Participant's CD4^+^ T-cell counts were enumerated at 6-monthly intervals for up to 7 years. Sixteen participants reported ART use during the course of observation and their data was truncated at the point of ART initiation. All data reported here is therefore based on ART-naïve participants. The Uganda Virus Research Institute Science and Ethics Committee as well as the National Council of Science and Technology approved the study. All subjects provided written informed consent for participation. After classification of the participants, blood specimens were collected to concurrently evaluate the HIV viral load, infecting clade and HIV-specific IFN-γ response. Participant demographics are illustrated in tables 1 and 2.

### Stratification of participants into HIV disease progression groups

Six-monthly CD4 T-cell counts were used to compute CD4 T-cell slopes in order to stratify the participants into discrete HIV disease progression groups (table 1). Disease progression was estimated using a random effects multilevel regression model. Individual slopes of CD4 T-cells were estimated simultaneously for all patients. CD4 decline varies across time, generally being steeper in the early stages and less steep at later stages. The slope therefore changes over time. To estimate the CD4 curve, we used all CD4 counts available, including those that were taken up to 6 years before 2002. Blood for CD4 slopes evaluation was drawn from 1996 to 2006, blood for IFN-γ evaluations was drawn from 2002. In order to assess the CD4 decline, we included a term in the statistical multilevel model for pre/post 2002 CD4 counts. Once a curve was determined, we then estimated the average post-2002 slope by taking two slope points on the curve and averaging them. The two slope points taken were: the estimate of the slope during the first month of 2002, and the estimate of the slope during the first month of 2006 (4 years later). Some participants did not have a CD4 data point in 2006. However, the estimated slope varies across time, so in order that the estimate be consistent across all participants, we used the regression coefficients to estimate what the slope in the first month of 2006 would be. For some participants, their baseline CD4 T-cell count was taken early in the course of their disease progression; while for others, their baseline CD4 count was taken later in the course of their disease progression. To control for the stage of disease progression at baseline, the multilevel regression model included an interaction term between baseline CD4 and time. In order to determine progression groups, the CD4 decline was examined, controlling for both the baseline CD4 and age of the participant. Gender was not included in the final model because it has already been shown that gender had no significant impact on CD4 decline in this population (personal communication from Leigh Anne Shafer). CD4 decline outliers on the fast progression tail of the normal distribution curve were classified as rapid progressors (RP; [stratification group1]), table 1. Outliers on the slow progression tail of the normal distribution curve were classified as slow progressors (SP; [stratification group 5]), table 1. Rather than use an absolute cut-off, such as the 95^th^ percentile, outliers were determined visually. That is, the steepness of the 110 CD4 declines was on a continuum, and the rapid and slow progressors were determined by a reasonable gap in the continuum.

After visual inspection for a distinct gap in the continuum, disease progression groups were determined. RPs were those with CD4 slopes less than −101 cells per µl/year; NPs had CD4 slopes between −91 and +10; SPs had CD4 slope that were rising by at least 16 cells per µl/year. Because the participants were stratified by CD4 slope changes, the parameters evaluated in these groups would be distributed as a continuum spectrum where the difference between two adjacent groups is not significant but there would be significant difference between the RP and SP extreme groups. Consequently, 14 SP and 7 RP derived from the two extreme groups were selected for subsequent evaluation of the relationships between HIV-induced T-cell response and disease progression.

While individual CD4 measurements may include errors and random noise, the multilevel model helped to overcome this by utilising CD4 counts from all study participants in order to estimate the individual participant CD4 slope. This method was particularly important for participants on whom very few CD4 measurements were available. Without the multilevel regression modelling approach, an estimated slope obtained from only a few CD4 T-cell counts would be very unreliable; the multilevel modelling approach took this into account by reducing the weight assigned to individuals with few CD4 count measurements and bringing their estimated slope closer to the mean slope across all individuals. Using all participants' CD4 counts, an estimate of the variance in CD4 slope across participants was obtained. By combining individual 6-monthly interval CD4 measurements with the group variance, the multilevel regression model obtained the best estimate of CD4 slope for each individual participant resulting in distinct stratification into rapid and slow progressors.

The average baseline CD4 T-cell counts, on or after the date of the specimen used to assess viral load, infecting clade and HIV-specific IFN-γ response, across all 110 participants, was approximately 500 cells/µL; this was assumed to be the cohort starting CD4 T-cell count for each participant, after determining their individual regression coefficients for CD4 decline. Because CD4 decline varies across time, average CD4 decline was computed by assessing the decline at two points and then averaging them. The two points were enrolment and 48 months post-enrolment, assuming a constant starting CD4 T-cell count of 500 cells/µL for each person. Based on evidence from this and other HIV cohorts [58], [59], we square root transformed CD4 counts in the multilevel regression analysis in order to attain normal distribution.

### Plasma viral load, CD4 T-cell counts

HIV-1 RNA plasma viral load and CD4 counts were quantified using the HIV-1 RNA 3.0 bDNA assay (Bayer™), and FACScount (Beckton Dickinson™) respectively according to the manufacturer's protocols. The threshold for RNA detection was 50 copies/ml. All plasma viral loads are presented as log10 transformed data. Four digit high resolution Characterisation of the infecting viral subtype was based on the Gag sequences as described below.

### HLA typing

HLA tissue typing was initially performed at low/medium resolution using in-house polymerase chain reaction-sequence specific primers (PCR-SSP). High resolution HLA class I typing was perfomed by PCR using reference strand conformational analysis (RSCA) as previously described [60]. Briefly, locus specific primers were used to amplify HLA-A,-B and -C loci using PCR.The PCR product were hybridised to fluorescently labelled refrence strands (FLR's) to form heteroduplexes. These heteroduplexes were run on a non-denaturing polyacrylamide gel with a laser based fluorescence detection system. Heteroduplex mobility values differed depending on the similarity of the PCR product with the labelled reference strand. HLA types were assigned by comparing the obtained mobility values with known values for different alleles.

### Sequencing of the HIV Gag region

RNA, extracted from either plasma or serum, was subjected to reverse transcription and PCR (RT-PCR), and an approximately 720 bp region of the gag gene encompassing the p17/p24 junction was amplified by nested PCR [61]. PCR products were then sequenced on an ABI 377 automated sequencer according to manufacturer's instructions (ABI, Warrington, UK). Sequences thus obtained were aligned with homologous regions from reference viral strains obtained from the Los Alamos Database (http://hiv-web.lanl.gov), using the BioEdit package (http://www.mbio.ncsu.edu). Neighborhood joining phylogenetic trees were constructed using the Treecon package [62], employing a Kimura distance matrix. Each virus was assigned a subtype by comparison of its sequence with reference strains. Sequences were also examined for the presence of inter-subtype recombinants, which are common in this population, by employing the Simplot programme as described in [63] and http://www.welch.jhu.edu/~sray.

### HIV peptides and preparation of pools

HIV peptides were obtained through the National Institute of Health, AIDS Research and Reference Reagent programme (https://www.aidsreagent.org/Index.cfm). The 20 amino acid peptides with 10-amino acid overlaps between sequential peptides were based on consensus sequences of clades A, B, C and D Gag protein and Clade B Nef, Tat, Vif, Rev, Vpr, Vpu and Pol proteins. For the Gag region only, we evaluated relationships between cross-clade recognition and HIV disease progression.

### ELISpot protocol

Peripheral blood mononuclear cells (PBMCs) were isolated from whole-heparinised blood using Ficoll Histopaque (Sigma) density gradient centrifugation. ELISpot assays were performed as previously described [33], [64] with some slight modifications. Briefly, an ELISpot screening assay using matrices of 10 pooled peptides was performed with each individual peptide appearing twice in separate pools within the matrix. A PBMC specimen was considered positive if it had a positive response in both matrix pools that shared the peptide and this was subsequently confirmed using individual peptides. Freshly isolated PBMCs were plated in duplicate at 50,000–200,000 cells per well and incubated with peptide pools at a final concentration of 2 µg/ml per peptide for 16–18 hours at 37°C in a 5% CO_2_ atmosphere. Six negative control wells (cells and media) as well as three positive control wells (2 µg/ml PHA) were also included on the plate. Spots were developed using the Vectastain Elite ABC kit and Vector Novared substrate kit (Vector Laboratories Inc.) according to the manufacturer's instructions. Spots were counted using a KS ELISpot image analyser (Carl Zeiss), and were expressed as number of spot forming units (SFU) per million input PBMCs. A test was regarded as positive when the response was ≥50 SFU/10^6^ PBMC and at least twice the mean background (6 wells of cells and media) response. All ELISpot data is presented herein as net response after having subtracted all the background responses. All net responses below 50 SFU/million PBMCs were assumed to equate to zero response in all statistical analyses and graphical representations

For each evaluated HIV Gag, Nef, Tat, Vif, Rev, Vpr, Vpu and Pol protein, the frequency of HIV-specific IFN-γ responders was evaluated as the proportion of individuals within a stratification group responding to that protein with induction of IFN-γ.

### Statistical Analysis

Participants were stratified according to HIV-1 disease progression groups using a multilevel regression analysis model. As the untransformed CD4 count data was not normally distributed, medians and interquartile ranges were used for the summary presentations. Plasma viral load data was log-transformed in order to normalize it for subsequent analysis and was analysed using unpaired the Students t-test. The Kruskal-Wallis rank test was used to compare the median CD4 counts and IFN-γ responses between the groups. Spearman rank correlation coefficients were used to demonstrate correlations between CD4 cell declines and HIV-induced T-cell responses. Categorical data was compared using the two-tailed Fisher's exact test. Ms Excel and Graph Pad 4.0 were used for all the graphical presentations. All analyses were performed using Stata v 9.0 (Stata Corp, Texas).
